# Prophylactic endotracheal intubation in critically ill patients with upper gastrointestinal bleed: A systematic review and meta‐analysis

**DOI:** 10.1002/jgh3.12195

**Published:** 2019-05-24

**Authors:** Dipayan Chaudhuri, Kirles Bishay, Parul Tandon, Vatsal Trivedi, Paul D James, Erin M Kelly, Kednapa Thavorn, Kwadwo Kyeremanteng

**Affiliations:** ^1^ Department of Critical Care McMaster University Hamilton Ontario Canada; ^2^ Department of Gastroenterology University of Toronto Toronto Ontario Canada; ^3^ Department of Anesthesiology and Pain Medicine The Ottawa Hospital, University of Ottawa Ottawa Ontario Canada; ^4^ Department of Medicine The Ottawa Hospital, University of Ottawa Ottawa Ontario Canada; ^5^ Ottawa Hospital Research Institute The Ottawa Hospital Ottawa Ontario Canada; ^6^ School of Epidemiology, Public Health and Preventative Medicine, Faculty of Medicine University of Ottawa Ottawa Ontario Canada; ^7^ Division of Palliative Care The Ottawa Hospital, University of Ottawa Ottawa Ontario Canada; ^8^ Division of Critical Care The Ottawa Hospital, University of Ottawa Ottawa Ontario Canada; ^9^ Institute du Savoir Montfort Ottawa Ontario Canada

**Keywords:** intensive care unit, meta‐analysis, prophylactic intubation, upper gastrointestinal bleed

## Abstract

**Background and Aim:**

Prophylactic endotracheal intubation for airway protection prior to endoscopy for the management of severe upper gastrointestinal bleeding (UGIB) is controversial. The aim of this meta‐analysis is to examine the clinical outcomes and costs related to prophylactic endotracheal intubation compared to no intubation in UGIB.

**Methods:**

EMBASE, MEDLINE, and the Cochrane Central Register of Controlled Trials were used to identify studies through June 2017. Data regarding mortality, total hospital and intensive care unit length of stay (LOS), pneumonia, and cardiovascular events were collected. The DerSimonian‐Laird random effects models were used to calculate the inverse variance‐based weighted, pooled treatment effect across studies.

**Results:**

Seven studies (five manuscripts and two abstracts) were identified (5662 total patients). Prophylactic intubation conferred an increased risk of death (odds ratio [OR], 2.59, 95% confidence interval [CI]: 1.01–6.64), hospital LOS (mean difference, 0.96 days, 95% CI: 0.26–1.67), and pneumonia (OR 6.58, 95% CI: 4.91–8.81]) compared to endoscopy without intubation. The LOS‐related cost was greater when prophylactic intubation was performed ($9020 per patient, 95% CI: $6962–10 609) compared to when it was not performed ($7510 per patient, 95% CI: $6486–8432). There was no difference in risk of cardiovascular events after sensitivity analysis.

**Conclusion:**

Prophylactic intubation in severe UGIB is associated with a greater risk of pneumonia, LOS, death, and cost compared to endoscopy without intubation. Randomized trials examining this issue are warranted.

## Graphical abstract

Prophylactic intubation in severe upper gastrointestinal bleeding may be associated with greater risk of pneumonia, length of stay, death, and cost compared to endoscopy without intubation. Randomized trials examining this issue are warranted.

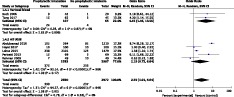

## Introduction

Upper gastrointestinal bleeds (UGIB) are a relatively common presentation to hospitals, with an overall incidence of 50–172 per 100 000 people,^1^, ^2^ Esophagogastroduodenoscopy (EGD) may lead to an increase in respiratory‐related adverse events such as severe aspiration and pneumonia in massive UGIB.^3^ The main goals of prophylactic intubation are to ensure airway protection during deep sedation and to reduce aspiration risk. Despite a paucity of high‐quality evidence, current guidelines recommend prophylactic intubation prior to endoscopy for UGIB among critically ill patients.^4^

Previous studies have demonstrated an increased risk of pneumonia with prophylactic intubation in UGIB compared to no intubation.^5^ Although studies examining differences in mortality, length of stay (LOS) in hospital, intensive care unit (ICU) LOS, pneumonia, and cardiovascular events have suggested an increased risk of these outcomes, they have been limited by small sample size, and their study design vary. The increased costs associated with prophylactic intubation for endoscopic management of UGIB have not previously been described.

The aim of this study was to review the existing literature to estimate the risk of cardiovascular events, risk of pneumonia, increased LOS, and risk of death associated with prophylactic intubation for the management of UGIB in critically ill patients.

## Methods

The Preferred Reporting Items for Systematic Reviews and Meta‐Analyses (PRISMA) statement guidelines were used to conduct the systematic review and meta‐analysis.^6^ The study followed an a priori established protocol.

### *Data sources and search strategy*

We searched EMBASE, MEDLINE, and the Cochrane Central Register of Controlled Trials for relevant studies to identify all studies that compared prophylactic endotracheal intubation to no intubation for the medical management of adult patients with UGIB using the following key word themes: (i) *endotracheal intubation* (intratracheal intubation or endotracheal intubation) and (ii) *gastrointestinal bleeding* (gastrointestinal bleeding or gastrointestinal hemorrhage or [stomach, gastric, gastrointestinal, gi, duodenal, gastroduodenal, peptic, esophageal, oesophageal, varices, or variceal] and [haemorrhage, hemorrhage, bleed, rebleed or blood loss]). The search was conducted from May 2017 to June 2017. A health sciences librarian with expertise in systematic reviews developed the specific search strategy used with the input of the project team. A copy of the search strategy is included in the Appendix S1, Supporting information. The bibliographies of included articles and similar systematic reviews were also examined. Finally, we contacted experts in the field to identify any additional studies and gray literature that should be considered in this review. An updated version of this search was conducted in May 2018.

### *Selection criteria*

We included studies that (i) included all patients older than 16 years of age undergoing EGD for severe UGIB (defined as patients who needed immediate endoscopy or admission to an ICU for the management of UGIB); (ii) compared prophylactic intubation to no prophylactic intubation; and (iii) reported on at least one of the following outcomes: cardiac events, pneumonia, LOS (in hospital and ICU), and death.

### *Data extraction and collection*

Two reviewers (Dipayan Chaudhuri and Kirles Bishay) independently reviewed the titles and abstracts to identify articles for full‐text review. Any discrepancies in the inclusion of abstracts between reviewers were settled through consensus. Data were extracted for all studies using a standardized data collection form devised by Dipayan Chaudhuri and Kirles Bishay, which included study design; inclusion and exclusion criteria; and a priori selected outcomes, including mortality, hospital LOS, ICU LOS, pneumonia, and cardiovascular events.

### *Study outcomes*

The primary outcomes of our analysis were the risk of in‐patient mortality and hospital LOS. Secondary outcomes included pneumonia, cardiovascular events (composite outcome of myocardial infarction and cardiac arrests), and ICU LOS.^7^, ^8^, ^9^ We also performed a priori subgroup analysis based on the etiology of the UGIB. We stratified studies based on whether they looked at only variceal UGIB *versus* all UGIB, including variceal bleeds, as none of the included studies analyzed nonvariceal bleeds alone.

### *Cost analysis*

To approximate the resource use associated with prophylactic intubation in UGIB, we performed a cost analysis based on ICU LOS and hospital LOS. We derived ICU and hospital costs using the methodology and average costs reported by Kahn *et al.* 10 For ICU costs, the daily direct costs in United States dollars were estimated as follows: ICU day 1 was $3678, day 2 $1057, day 3 $839, day 4 $834, and for each day beyond day 4 was $690 per day. The estimated hospital LOS cost was $249 per day. We applied direct variable costs, which exclude salaries, equipment, and other fixed infrastructure costs as this reflects the direct and immediate financial implications related to LOS. Costs were adjusted to 2017 using a consumer price index reported by the US Bureau of Labor Statistics.^11^

### *Methodological quality assessment*

The National Institute of Health's quality assessment tools^12^ for case–control and cohort studies were used to evaluate the studies included. Two reviewers (Dipayan Chaudhuri and Kirles Bishay) evaluated each study independently, and discrepancies were resolved through consensus between the reviewers. The overall quality of evidence was assessed using the Grades of Recommendation, Assessment, Development and Evaluation (GRADE) approach.^13^

### *Statistical analysis*

Review Manager version 5.3 (Cochrane Collaboration Review manager) was used for statistical analysis.^14^ The DerSimonian‐Laird random effects model was used to calculate the inverse variance‐based weighted, pooled treatment effect across studies. Odds ratios (OR) were calculated for all primary and secondary outcomes using random effects models. A sensitivity analysis was also performed, excluding studies deemed to be of lower quality. We measured heterogeneity across studies using the χ^2^ test (with a *P* < 0.10 denoting significant heterogeneity) and the *I* ^2^ statistic (with a value >50% denoting significant heterogeneity) as described by Deeks and Higgins.^15^ Statistical significance was defined as a *P* < 0.05.

## Results

### *Study selection*

**Figure 1:**
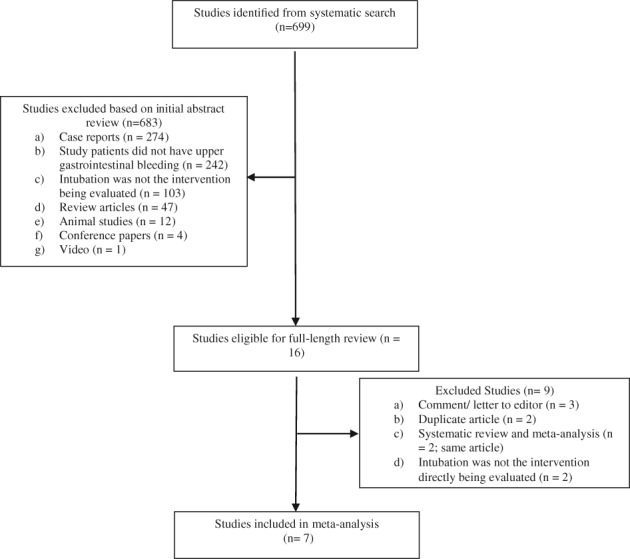
Flow diagram for study selection. Studies were selected through a two‐step process consisting of (i) title and abstract review followed by (ii) full‐text review of selected studies.

The initial literature search resulted in 980 articles (301 from MEDLINE, 633 from EMBASE, and 37 from Cochrane [central]), of which 699 nonduplicate articles were selected for abstract review. Fourteen abstracts were selected for manuscript evaluation. Of these, seven met the inclusion criteria and were included in our analysis.^7^, ^8^, ^16^, ^17^, ^18^, ^19^, ^20^ The coefficient of agreement between reviewers was 0.85 (95% confidence interval [CI]: 0.71–0.98). Figure 1 illustrates our study flow diagram. The included studies were published from 2006 to 2017. Five studies were full‐length manuscripts,^7^, ^8^, ^17^, ^18^, ^19^ and two were abstracts.^16^, ^20^ All studies were retrospective. Study characteristics are provided in Table S1. We classified three studies as low quality.^16^, ^19^, ^20^ Lohse *et al.* 19 had significantly different baseline characteristics between their two groups. The studies of Abdulsamad *et al.* 16 and Perisetti *et al.* 20 were abstract only publications. An updated search in May 2018 yielded no new studies.

### *Primary outcomes*

**Figure 2:**
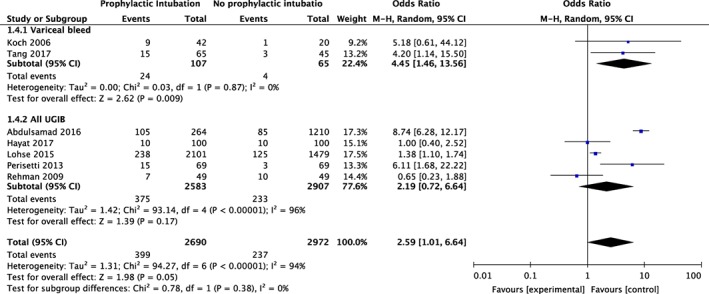
Meta‐analysis of mortality and prophylactic intubation in upper gastrointestinal bleeding (UGIB) stratified by variceal bleed only and all UGIB. CI, confidence interval(s); M‐H, mantel–Haenszel odds ratio.

Overall, 5662 patients with UGIB were included in the analysis. The combined in‐hospital mortality was 14.8% (399/2690) for the prophylactic intubation group and 8.0% (237/2972) for the no intubation group. Overall, prophylactic intubation was associated with increased mortality compared to no intubation (OR 2.59; [95% CI: 1.01–6.64], *I* ^2^ = 94%). Subgroup analysis was performed based on the specific etiology of UGIB: variceal bleed *versus* all UGIB. All UGIB encompasses both variceal and nonvariceal bleeds as no study explored nonvariceal bleeding alone. Prophylactic intubation conferred increased mortality on patients presenting with variceal bleed (OR 4.45; [95% CI: 1.46–13.56]) but did not affect survival in all patients with UGIB (OR 2.19; [95% CI: 0.72–6.64]). There was no heterogeneity in the variceal group (*I* ^2^ = 0%) and high heterogeneity in the UGIB group (*I* ^2^ = 96%) (Fig. 2). These results were largely maintained in sensitivity analysis (Fig. S4). The odds of mortality in the overall UGIB prophylactic intubation group was attenuated but less heterogeneous (OR 1.57 [95% CI: 0.61–4.04], *I* ^2^ = 56%) and remained insignificant in the all UGIB group (OR 0.83; [95% CI: 0.41–1.67]) with no heterogeneity. There were no changes in the OR or the heterogeneity of the variceal subgroup.

**Figure 3:**
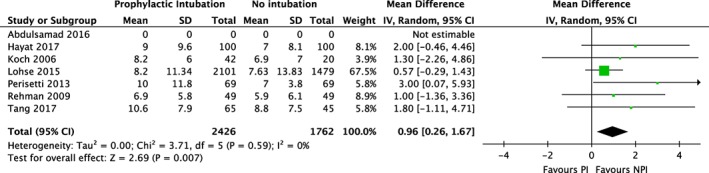
Meta‐analysis of hospital length of stay and prophylactic intubation in upper gastrointestinal bleeding. CI, confidence interval(s); IV, inverse variance.

The hospital LOS was higher in the prophylactic intubation group (mean difference [MD], 0.96 days; [95% CI: 0.26–1.67]). There was no heterogeneity between studies (*I* ^2^ = 0) (Fig. 3). Sensitivity analysis (Fig. S3) showed an increase in MD (1.52 days, [95% CI: 0.16–2.88]) with no change in heterogeneity.

### *Secondary outcomes*

**Figure 4:**
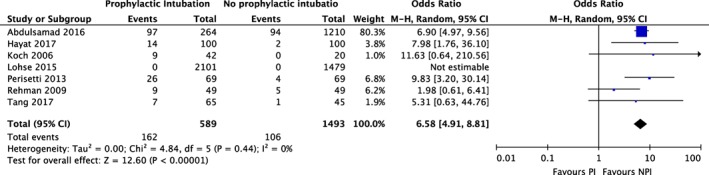
Meta‐analysis of rates of pneumonia and prophylactic intubation in upper gastrointestinal bleeding. CI, confidence interval(s); M‐H, mantel–Haenszel odds ratio.

**Figure 5:**
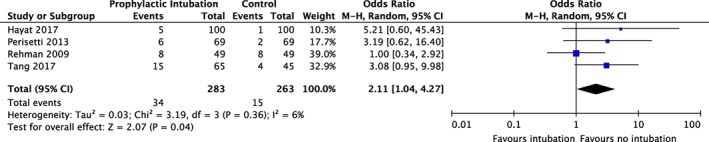
Meta‐analysis of cardiac complications and prophylactic intubation in upper gastrointestinal bleeding. CI, confidence interval(s); M‐H, mantel–Haenszel odds ratio.

The prophylactic intubation group demonstrated significantly higher rates of pneumonia (OR 6.58; [95% CI: 4.91–8.81], *I* ^2^ = 0%) (Fig. 4). Sensitivity analysis (Fig. S2) attenuated the effect (OR 3.95; [95% CI: 1.75–8.94]), with no change in heterogeneity. The odds of the composite outcome of cardiac complications (Fig. 5) was also higher in the prophylactic intubation group (OR 2.11; [95% CI: 1.04–4.27], *I* ^2^ = 6%), with a similar OR following sensitivity analysis, although this no longer remained significant (OR 2.03, [95% CI: 0.80–1.75], *I* ^2^ = 31%) (Fig. S5). There was also a trend toward increased ICU LOS (Table S1) in the prophylactic intubation group with high heterogeneity (MD, 1.21; [95% CI: −0.20, 2.62], *I* ^2^ = 69%). Sensitivity analysis (Fig. S6) showed a lower MD with improved heterogeneity (MD 0.48; [95% CI: 0.01–0.96], *I* ^2^ = 0).

### *Cost analysis*

While there was no significant difference in ICU LOS between the two groups, there was a trend toward increased ICU LOS in the prophylactic intubation group. This was reflected in the overall costs of ICU stay in both groups: the prophylactic intubation group incurred a cost of $7778 per patient in the ICU (95% CI: 5229–9859) compared to $6180 (95% CI: 4985–7248) in the no intubation group, a MD of $1598 (95% CI: 1310–1621). When examining total direct variable hospital costs, this trend was maintained. The prophylactic intubation group incurred costs of $9020 per patient (95% CI: 6962–10 609) compared to $7510 (95% CI: 6486–8432) in the no intubation group, with a MD of $1510 (95% CI: 1370–1632).

### *Methodological quality and risk for bias*

The quality of evidence on the use of prophylactic intubation varied from good to indeterminate (Table S2–S8). Most of the studies included were of moderate to good quality. Three of the studies were rated as moderate quality as they did not include justification for study sample size or matched controls. However, only one of the studies that did not have matched controls had significantly different baseline characteristic between the intervention and control groups. Both included abstracts were of indeterminate quality due to insufficient information present in the abstract. The overall quality of evidence on the topic based on outcome varied from moderate to very low. For in‐hospital mortality and cardiac complications, the quality of the evidence was low (+2 according to the GRADE approach). For the rate of pneumonia, the quality of the evidence was moderate (+3 according to the GRADE approach). The quality of the evidence for hospital LOS and ICU LOS was very low (+1 according to the GRADE approach) due to small effect sizes and heterogeneity in the results for ICU LOS (Table S9).

## Discussion

In this systematic review and meta‐analysis, an increased rate of mortality was found among ICU patients who were prophylactically intubated prior to EGD for severe UGIB, primarily driven by patients with variceal bleeds. Hospital LOS was longer in the intubated group. Finally, among those prophylactically intubated, there were increased rates of pneumonia compared to those who were not intubated.

The practice of prophylactically intubating patients for the endoscopic management of severe UGIB varies significantly, and this may be related to endoscopist experience and patient factors.^21^ A stable airway may allow for ease of intervention on bleeding lesions; however, the evidence regarding the efficacy of intubation for reducing endoscopy‐related aspiration events is unclear.^22^ Furthermore, the potential outcomes associated with prophylactic intubation should be considered.^22^ This review highlights important increased risks of death and pneumonia associated with prophylactic intubation, as well as the related increases in LOS and hospital costs.

One previous systematic review and meta‐analysis examined the impact of prophylactic intubation before EGD on UGIB.^5^ The authors found a statistically higher rate of pneumonia for patients who were prophylactically intubated but found no differences in mortality. However, the study included four articles (two were abstracts) and examined 367 patients. Since the publication of this review, multiple large retrospective studies have been conducted on the topic. Our review builds on work previously performed in this field to include over 5000 patients and considers numerous relevant outcomes. To our knowledge, this is also the first study to present a cost analysis regarding the impact of prophylactic intubation in severe UGIB.

Endotracheal intubation carries significant risk, particularly in critically ill patients. A national audit project in the United Kingdom (NAP4) examined and reported adverse events during airway management in the NAP4.^23^ While immediate procedure‐related adverse events are uncommon, approximately 25% of all adverse events occurred in the ICU setting periintubation.^22^ In patients with UGIB, many practitioners classically use rapid sequence induction (RSI) techniques to reduce the risk of aspiration. Critically ill patients with UGIB are often hemodynamically unstable, resulting in a consistently high mortality despite advancements in therapy.^22^ The NAP4 identified the use of RSI in the ICU as a major cause of concern secondary to administration of preselected doses of induction agents in patients with unstable hemodynamics, a population inclusive of critically ill UGIB patients. Finally, aspiration during airway management was the most common cause of death in the NAP4, and patients with UGIB would certainly be at increased risk.

The results of this review are consistent with what would be expected from the NAP4 study. Although there was significant heterogeneity among studies, prophylactic intubation showed increased mortality compared to no intubation. While most studies showed a trend toward increased mortality,^7^, ^8^, ^16^, ^17^, ^18^, ^19^, ^20^ none showed a statistically significant increase in mortality between groups. As all studies were retrospective, it is difficult to definitively state causality for the increase in mortality; however, prophylactic intubation was also associated with significantly higher rates of pneumonia and a trend toward increased cardiac complications [cardiac arrest, myocardial infarction (MI)]. Therefore, it is plausible that the hemodynamic compromise and aspiration risks associated with tracheal intubation and ongoing mechanical ventilation could be responsible for the increased mortality observed, particularly in variceal bleeds, as cirrhotic patients in the ICU tend to be sicker and relatively immunocompromised compared to noncirrhotic patients. The high mortality found in variceal bleeds is also consistent with previous data suggesting that cirrhotic patients have worse clinical outcomes and increased mortality independent of the severity of illness, leading to ICU stay.^24^, ^25^

The cost analysis demonstrates a trend toward increased costs in the prophylactic intubation group secondary to the increased hospital LOS. On average, there appears to be a difference of approximately $1510 USD (95% CI: 1370–1632) between the two groups based on LOS alone. Notably, the results of the cost analysis were preliminary as it only captured ICU and hospital costs. The unit costs of the ICU and hospital were also obtained from a single US study, which might not be able to generalize to other settings. Future research should perform a full economic evaluation of and include both upfront and downstream costs and health outcomes of intubation.

### *Study limitations*

This review adds to the current literature in a significant way. There are relevant findings in multiple important clinical outcomes, and this is the largest meta‐analysis on this topic, including over 5600 patients. However, there are a few limitations to this study. A primary limitation of this review is the small number of studies included. Only five full‐text articles and two abstracts sufficiently addressed the research question, and this may result in potential bias in heterogeneity estimates, particularly in some of the secondary analyses where a smaller number of studies were included as all studies did not report the same data. Furthermore, two of the studies^16^, ^19^ accounted for over 50% of the review population. While this has the potential to bias some of the results, particularly in terms of pneumonia and hospital LOS, results from the other studies are similar in magnitude and effect, and thus, the potential for inherent bias is low. This is further supported by the lack of heterogeneity for these outcomes. In addition, this limitation was addressed by performing sensitivity analyses, which excluded three studies,^16^, ^19^, ^20^ and maintained that, in general, prophylactic intubation leads to worse outcomes and longer LOS. Moreover, given the dichotomous nature of the intervention (intubation or no intubation), there was little variability between studies in methodology, adding certainty to the results.

The retrospective nature of the studies included also prevented the selection of study populations that are identical at baseline and avoided introducing bias regarding case selection, outcome selection, and confounding. Specifically, it is plausible that there is an inherent difference between patients who were chosen to be prophylactically intubated and those who were not intubated. A key variable that was not captured in these studies was hemodynamic status at the time of intubation. In addition, the reviewed papers did not provide any insight into whether patients were unnecessarily intubated based on the absence of endoscopic findings or intervention. However, in the majority of studies^7^, ^8^, ^16^, ^17^, ^18^, ^20^ included, there does not appear to be significant differences in the reported baseline characteristics between groups, with two studies confirming this through propensity matching.^8^, ^17^ One large study did not perform matching and did have significant differences in baseline characteristics between the two study groups.^19^ However, while the prophylactic intubation group in that study had a more severe shock, they were also younger and had lower scores on the Charlson Comorbidity Index (CCI). As a result, it cannot be concluded that the prophylactic intubation group in that study was at higher risk for pneumonia, cardiac complications, mortality or prolonged hospital or ICU LOS.

Using the GRADE approach to assess the quality of systematic reviews, the quality of evidence in this study varied based on the outcome being examined. While some of the effect sizes were quite large, all studies included were observational, and the lack of propensity matching in all but two studies may have introduced potential confounding factors.

In conclusion, prophylactic intubation prior to EGD in critically ill patients with UGIB is associated with an increase in mortality, particularly in patients with variceal bleeds, although these data are heterogeneous and may be confounded by factors not addressed in individual studies. It is also associated with higher rates of pneumonia. Furthermore, there is a trend toward increased hospital and ICU LOS, cardiac complications, and increased cost. A comprehensive review of adverse events related to prophylactic intubation in UGIB should be pursued as this topic remains controversial.

## Supporting information


**Appendix S1** Search strategy used for systematic review across MEDLINE, EMBASE, and COCHRANE CENTRAL REGISTER OF CONTROLLED TRIALS.

**Figure S1** Meta‐Analysis of ICU LOS and prophylactic intubation in UGIB. CI, confidence interval(s); IV, inverse variance.

**Figure S2** Sensitivity analysis pneumonia.

**Figure S3** Sensitivity analysis hospital LOS.

**Figure S4** Sensitivity analysis mortality.

**Figure S5** Sensitivity analysis cardiac.

**Figure S6** Sensitivity analysis ICU LOS.

**Table S1** Baseline characteristics of studies.

**Table 2–8** Quality assessment of Individual Studies using the National Institute of Health's quality assessment tools for case–control and cohort studies.
**Table S9** Overall assessment of the quality of evidence presented in the systematic review using the GRADE approach.

## References

[1] Mokhtare M , Bozorgi V , Agah S *et al.* Comparison of Glasgow‐Blatchford score and full Rockall score systems to predict clinical outcomes in patients with upper gastrointestinal bleeding. Clin. Exp. Gastroenterol. 2016; 9: 337–43. 27826205 10.2147/CEG.S114860 PMC5096755

[2] Longstreth GF . Epidemiology of hospitalization for acute upper gastrointestinal hemorrhage: a population‐based study. Am. J. Gastroenterol. 1995; 90: 206–10. 7847286

[3] Liebler JM , Benner K , Putnam T , Vollmer WM . Respiratory complications in critically ill medical patients with acute upper gastrointestinal bleeding. Crit. Care Med. 1991; 19: 1152–7. 1884614 10.1097/00003246-199109000-00010

[4] Ben‐Menachem T , Decker G , Early D *et al.* Adverse events of upper GI endoscopy. Gastrointest. Endosc. 2012; 76: 707–18. 22985638 10.1016/j.gie.2012.03.252

[5] Almashhrawi AA , Rahman R , Jersak ST *et al.* Prophylactic tracheal intubation for upper GI bleeding: a meta‐analysis. World J. Metaanal. 2015; 3: 4–10. 25741509 10.13105/wjma.v3.i1.4 PMC4346140

[6] Moher D , Liberati A , Tetzlaff J , Altman DG , Altman D . Preferred reporting items for systematic reviews and meta‐analyses: the PRISMA statement. PLoS Med. 2009; 6: e1000097. 19621072 10.1371/journal.pmed.1000097 PMC2707599

[7] Tang Y , Tang YM , Wang W . Prophylactic endotracheal intubation prior to urgent endoscopy in patients with suspected variceal hemorrhage: an evaluation of outcomes and complications. J. Gastroenterol. Hepatol. Res. 2017; 6: 2324–8.

[8] Rehman A , Iscimen R , Yilmaz M *et al.* Prophylactic endotracheal intubation in critically ill patients undergoing endoscopy for upper GI hemorrhage. Gastrointest. Endosc. 2009; 69: e55–9. 19481643 10.1016/j.gie.2009.03.002 PMC2737482

[9] Allescher H . Prophylactic endotracheal intubation for emergency endoscopy in critically ill patients? Gastroenterology. 2010; 138: 1627–9. 20175965 10.1053/j.gastro.2010.02.018

[10] Kahn JG , Kronick R , Kreger M , Gans DN . The cost of health insurance administration in California: estimates for insurers, physicians, and hospitals. Health Aff. 2005; 24: 1629–39. 10.1377/hlthaff.24.6.1629 16284038

[11] Bureau of Labor Statistics. *CPI Inflation Calculator* Cited 8 Jul 2017]. Available from URL: https://www.bls.gov/data/inflation_calculator.htm

[12] National Institutes of Health ‐ U.S. Department of Health and Human Services. *Background: Development and Use of Study Quality Assessment Tools ‐ NHLBI, NIH*, 2014. Cited 8 Jul 2017. Available from URL: https://www.nhlbi.nih.gov/health-pro/guidelines/in-develop/cardiovascular-risk-reduction/tools/background

[13] Guyatt GH , Oxman AD , Vist GE *et al.* GRADE: an emerging consensus on rating quality of evidence and strength of recommendations. BMJ. 2008; 336: 924–6. 18436948 10.1136/bmj.39489.470347.AD PMC2335261

[14] Review Manager (RevMan) [Computer Program]. Version 5.3. Copenhagen: The Nordic Cochrane Centre, The Cochrane Collaboration, 2014.

[15] Deeks J , Higgins J . Statistical Algorithms in Review Manager 5. Statistical Methods Group of the Cochrane Collaboration 2010 Cited 8 Jul 2017. Available from URL: https://training.cochrane.org/handbook/statistical-methods-revman5

[16] Abdulsamad M , Kamireddy C , Karki N , Sakam S , Kumar K , Okechukwu Ebiem AI . Should we intubate the patient first? outcomes of prophylactic endotracheal intubation for upper gastrointestinal bleeding. Am. J. Gastroenterol. 2016; 111: S1283.

[17] Hayat U , Lee PJ , Ullah H , Sarvepalli S , Lopez R , Vargo JJ . Association of prophylactic endotracheal intubation in critically ill patients with upper GI bleeding and cardiopulmonary unplanned events. Gastrointest. Endosc. 2017; 86: 500–509.e1. 28011279 10.1016/j.gie.2016.12.008

[18] Koch DG , Arguedas MR , Fallon MB , Koch DG , Arguedas MR , Fallon MB . Risk of aspiration pneumonia in suspected variceal hemorrhage: the value of prophylactic endotracheal intubation prior to endoscopy. Dig. Dis. Sci. 2007; 52: 2225–8. 17385037 10.1007/s10620-006-9616-0

[19] Lohse N , Lundstrøm LH , Vestergaard TR *et al.* Anaesthesia care with and without tracheal intubation during emergency endoscopy for peptic ulcer bleeding: a population‐based cohort study. Br. J. Anaesth. 2015; 114: 901–8. 25935841 10.1093/bja/aev100

[20] Perisetti A , Hasrat K , Abe S , William N , Ross M . Role of prophylactic pre‐esophagogastroduodenoscopy (EGD) endotracheal intubation (ETI) in upper gastrointestinal bleed (UGIB): a retrospective study. Am. J. Gastroenterol. 2013; 108: S15–6.

[21] Waye JD . Intubation and sedation in patients who have emergency upper GI endoscopy for GI bleeding. Gastrointest. Endosc. 2000; 51: 768–71. 10840326 10.1016/s0016-5107(00)70104-0

[22] Baradarian R , Ramdhaney S , Chapalamadugu R *et al.* Early intensive resuscitation of patients with upper gastrointestinal bleeding decreases mortality. Am. J. Gastroenterol. 2004; 99: 619–22. 15089891 10.1111/j.1572-0241.2004.04073.x

[23] Cook TM , Woodall N , Harper J , Benger J . Major complications of airway management in the UK: results of the Fourth National Audit Project of the Royal College of Anaesthetists and the Difficult Airway Society. Part 2: intensive care and emergency departments. Br. J. Anaesth. 2011; 106: 632–42. 21447489 10.1093/bja/aer059

[24] Rabe C , Schmitz V , Paashaus M *et al.* Does intubation really equal death in cirrhotic patients? Factors influencing outcome in patients with liver cirrhosis requiring mechanical ventilation. Intensive Care Med. 2004; 30: 1564–71. 15292984 10.1007/s00134-004-2346-x

[25] Juneja D , Gopal PB , Kapoor D , Raya R , Sathyanarayanan M . Profile and outcome of patients with liver cirrhosis requiring mechanical ventilation. J. Intensive Care Med. 2012; 27: 373–8. 21436171 10.1177/0885066611400277

