# Supplementary material for: Prophylactic endotracheal intubation in critically ill patients with upper gastrointestinal bleed: A systematic review and meta‐analysis
Source: JGH Open. 2019 May 24;4(1):22–8. doi: 10.1002/jgh3.12195 (PMC7008165; doi:10.1002/jgh3.12195)
Supplement: Supplementary file 1 — Appendix S1 Search strategy used for systematic review across MEDLINE, EMBASE, and COCHRANE CENTRAL REGISTER OF CONTROLLED TRIALS. [file JGH3-4-22-s001.docx]

Supplementary Materials Appendix 1

**Database: Ovid MEDLINE(R) Epub Ahead of Print, In-Process & Other Non-Indexed Citations, Ovid MEDLINE(R) Daily and Ovid MEDLINE(R) <1946 to Present> Search Strategy:**

--------------------------------------------------------------------------------

1 exp Intubation, Intratracheal/ (35715)

2 intubat*.tw,kw. (49265)

3 1 or 2 (67769)

4 Gastrointestinal Hemorrhage/ (39925)

5 ((stomach or gastric$ or gastrointestinal$ or gi or duoden$ or gastroduoden$ or peptic$ or esophag$ or oesophag$ or varices or variceal) adj3 (haemorrhag$3 or hemorrhag$3 or bleed$ or rebleed$ or blood loss)).tw. (38583)

6 4 or 5 (59051)

**7 3 and 6 (310)**

**Database: EBM Reviews - Cochrane Central Register of Controlled Trials <May 2017> Search Strategy:**

--------------------------------------------------------------------------------

1 exp Intubation, Intratracheal/ (3769)

2 intubat*.tw,kw. (9487)

3 1 or 2 (10711)

4 Gastrointestinal Hemorrhage/ (1248)

5 ((stomach or gastric$ or gastrointestinal$ or gi or duoden$ or gastroduoden$ or peptic$ or esophag$ or oesophag$ or varices or variceal) adj3 (haemorrhag$3 or hemorrhag$3 or bleed$ or rebleed$ or blood loss)).tw. (3826)

6 4 or 5 (4165)

**7 3 and 6 (37)**

**Database: Embase Classic+Embase <1947 to 2017 June 20> Search Strategy:**

--------------------------------------------------------------------------------

1 *intubation/ or *endotracheal intubation/ (20380)

2 intubat*.tw. (74405)

3 1 or 2 (79729)

4 *gastrointestinal hemorrhage/ or upper gastrointestinal bleeding/ (29244)

5 ((stomach or gastric$ or gastrointestinal$ or gi or duoden$ or gastroduoden$ or peptic$ or esophag$ or oesophag$ or varices or variceal) adj3 (haemorrhag$3 or hemorrhag$3 or bleed$ or rebleed$ or blood loss)).tw. (59147)

6 4 or 5 (69165)

**7 3 and 6 (633)**
