# Supplementary material for: Prophylactic endotracheal intubation in critically ill patients with upper gastrointestinal bleed: A systematic review and meta‐analysis
Source: JGH Open. 2019 May 24;4(1):22–8. doi: 10.1002/jgh3.12195 (PMC7008165; doi:10.1002/jgh3.12195)

Supplementary Figure 1: Meta-Analysis of ICU LOS and prophylactic intubation in UGIB. CI indicated confidence interval(s) and IV, inverse variance.

	Prophylactic Intubation			No intubation				Mean Difference
Study or Subgroup	Mean	SD	Total	Mean	SD	Total	Weight	IV, Random, 95% CI
Perisetti 2013	6	11.8	69	2	2.4	69	16.7%	4.00 [1.16, 6.84]
Rehman 2009	2.2	1.48	49	1.8	1.11	49	48.5%	0.40 [-0.12, 0.92]
Tang 2017	5.3	4	65	4.3	3.3	45	34.8%	1.00 [-0.37, 2.37]
**Total (95% CI)**			**183**			**163**	**100.0%**	**1.21 [-0.20, 2.62]**

Heterogeneity: $Tau^2 = 1.00$; $Chi^2 = 6.39$, df = 2 (P = 0.04); $I^2 = 69\%$
Test for overall effect: Z = 1.68 (P = 0.09)

Mean Difference
IV, Random, 95% CI

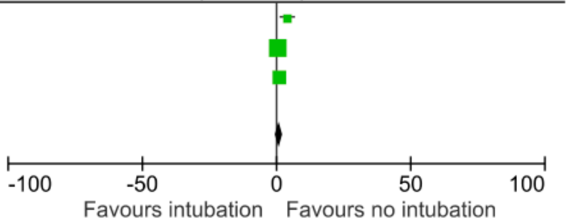

Supplement: Supplementary file 2 — Figure S1 Meta‐Analysis of ICU LOS and prophylactic intubation in UGIB. CI, confidence interval(s); IV, inverse variance. [file JGH3-4-22-s002.pdf]
