# Supplementary material for: Prophylactic endotracheal intubation in critically ill patients with upper gastrointestinal bleed: A systematic review and meta‐analysis
Source: JGH Open. 2019 May 24;4(1):22–8. doi: 10.1002/jgh3.12195 (PMC7008165; doi:10.1002/jgh3.12195)

Supplementary Figure 2: Sensitivity analysis for pneumonia and prophylactic intubation in UGIB. CI indicated confidence interval(s) and M-H, Mantel-Haenszel odds ratio.

	Prophylactic Intubation		No intubation			Odds Ratio
**Study or Subgroup**	**Events**	**Total**	**Events**	**Total**	**Weight**	**M-H, Random, 95% CI**
Abdulsamad 2016	97	264	94	1210	0.0%	6.90 [4.97, 9.56]
Hayat 2017	14	100	2	100	29.2%	7.98 [1.76, 36.10]
Koch 2006	9	42	0	20	7.9%	11.63 [0.64, 210.56]
Lohse 2015	0	2101	0	1479		Not estimable
Perisetti 2013	26	69	4	69	0.0%	9.83 [3.20, 30.14]
Rehman 2009	9	49	5	49	48.2%	1.98 [0.61, 6.41]
Tang 2017	7	65	1	45	14.6%	5.31 [0.63, 44.76]
**Total (95% CI)**		**256**		**214**	**100.0%**	**3.95 [1.75, 8.94]**
Total events	39		8			

Heterogeneity: $Tau^2 = 0.00$; $Chi^2 = 2.88$, df = 3 (P = 0.41); $I^2 = 0\%$

Test for overall effect: Z = 3.30 (P = 0.0010)

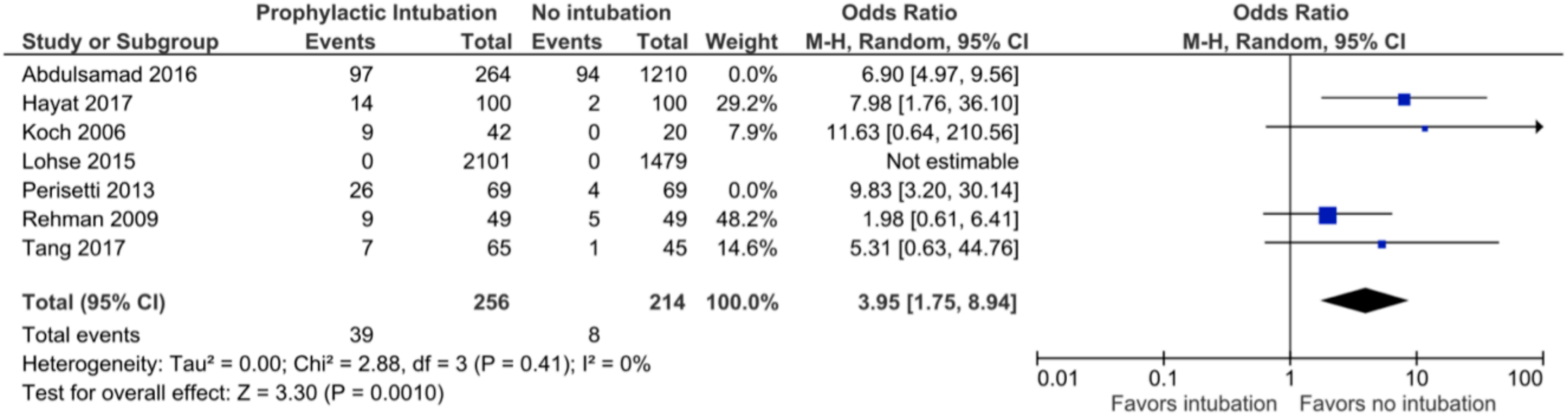

Supplement: Supplementary file 3 — Figure S2 Sensitivity analysis pneumonia. [file JGH3-4-22-s003.pdf]
