# Supplementary material for: Prophylactic endotracheal intubation in critically ill patients with upper gastrointestinal bleed: A systematic review and meta‐analysis
Source: JGH Open. 2019 May 24;4(1):22–8. doi: 10.1002/jgh3.12195 (PMC7008165; doi:10.1002/jgh3.12195)

Supplementary Figure 3: Sensitivity analysis hospital LOS and prophylactic intubation in UGIB. CI indicated confidence interval(s) and M-H, Mantel-Haenszel odds ratio.

	Prophylactic Intubation			No intubation				Mean Difference
**Study or Subgroup**	**Mean**	**SD**	**Total**	**Mean**	**SD**	**Total**	**Weight**	**IV, Random, 95% CI**
Abdulsamad 2016	0	0	0	0	0	0		Not estimable
Hayat 2017	9	9.6	100	7	8.1	100	30.5%	2.00 [-0.46, 4.46]
Koch 2006	8.2	6	42	6.9	7	20	14.5%	1.30 [-2.26, 4.86]
Lohse 2015	8.2	11.34	2101	7.63	13.83	1479	0.0%	0.57 [-0.29, 1.43]
Perisetti 2013	10	11.8	69	7	3.8	69	0.0%	3.00 [0.07, 5.93]
Rehman 2009	6.9	5.8	49	5.9	6.1	49	33.2%	1.00 [-1.36, 3.36]
Tang 2017	10.6	7.9	65	8.8	7.5	45	21.7%	1.80 [-1.11, 4.71]
**Total (95% CI)**			**256**			**214**	**100.0%**	**1.52 [0.16, 2.88]**

Heterogeneity: Tau² = 0.00; Chi² = 0.38, df = 3 (P = 0.94); I² = 0%
Test for overall effect: Z = 2.20 (P = 0.03)

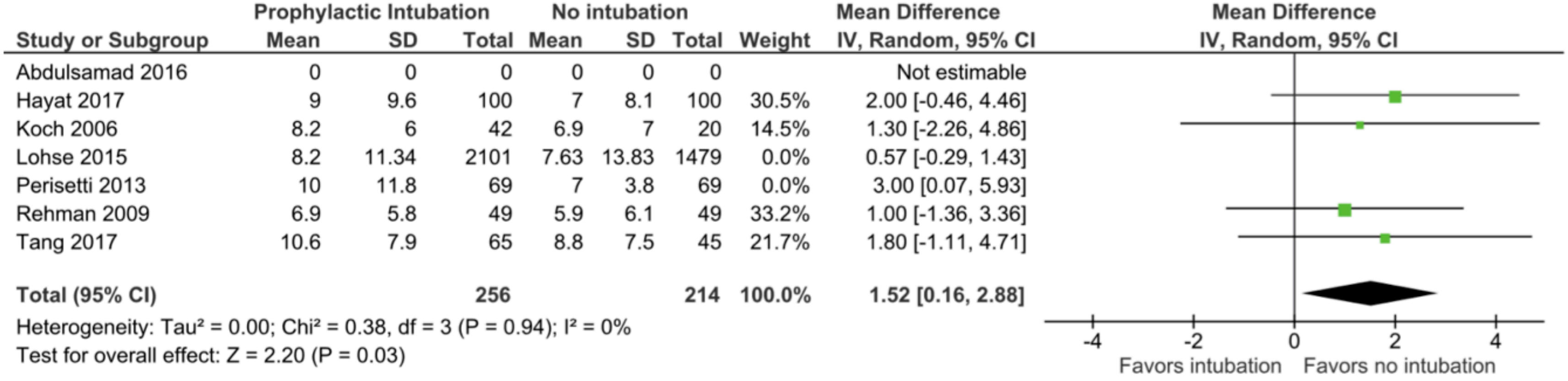

Supplement: Supplementary file 4 — Figure S3 Sensitivity analysis hospital LOS. [file JGH3-4-22-s004.pdf]
