# Supplementary material for: Prophylactic endotracheal intubation in critically ill patients with upper gastrointestinal bleed: A systematic review and meta‐analysis
Source: JGH Open. 2019 May 24;4(1):22–8. doi: 10.1002/jgh3.12195 (PMC7008165; doi:10.1002/jgh3.12195)

Supplementary Figure 4: Sensitivity analysis mortality and prophylactic intubation in UGIB. CI indicated confidence interval(s) and M-H, Mantel-Haenszel odds ratio.

	Prophylactic Intubation		No intubation			Odds Ratio
**Study or Subgroup**	**Events**	**Total**	**Events**	**Total**	**Weight**	**M-H, Random, 95% CI**
**1.4.1 Variceal bleed**						
Koch 2006	9	42	1	20	13.7%	5.18 [0.61, 44.12]
Tang 2017	15	65	3	45	24.7%	4.20 [1.14, 15.50]
**Subtotal (95% CI)**		**107**		**65**	**38.4%**	**4.45 [1.46, 13.56]**
Total events	24		4			
Heterogeneity: Tau² = 0.00; Chi² = 0.03, df = 1 (P = 0.87); I² = 0%						
Test for overall effect: Z = 2.62 (P = 0.009)						
**1.4.2 All UGIB**						
Abdulsamad 2016	105	264	85	1210	0.0%	8.74 [6.28, 12.17]
Hayat 2017	10	100	10	100	32.2%	1.00 [0.40, 2.52]
Lohse 2015	238	2101	125	1479	0.0%	1.38 [1.10, 1.74]
Perisetti 2013	15	69	3	69	0.0%	6.11 [1.68, 22.22]
Rehman 2009	7	49	10	49	29.4%	0.65 [0.23, 1.88]
**Subtotal (95% CI)**		**149**		**149**	**61.6%**	**0.83 [0.41, 1.67]**
Total events	17		20			
Heterogeneity: Tau² = 0.00; Chi² = 0.36, df = 1 (P = 0.55); I² = 0%						
Test for overall effect: Z = 0.52 (P = 0.60)						
**Total (95% CI)**		**256**		**214**	**100.0%**	**1.57 [0.61, 4.04]**
Total events	41		24			
Heterogeneity: Tau² = 0.50; Chi² = 6.77, df = 3 (P = 0.08); I² = 56%						
Test for overall effect: Z = 0.94 (P = 0.35)						
Test for subgroup differences: Chi² = 6.26, df = 1 (P = 0.01), I² = 84.0%						

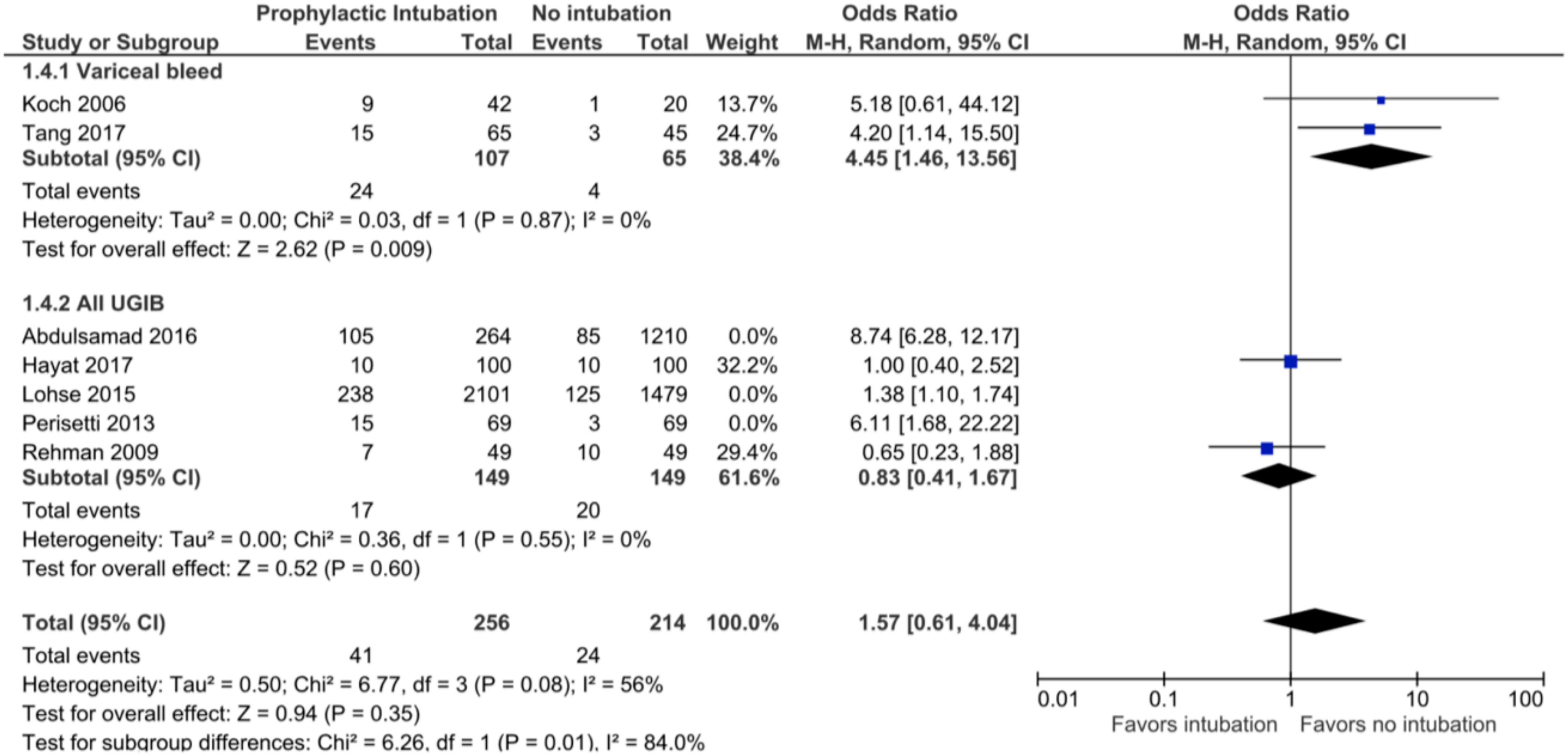

Supplement: Supplementary file 5 — Figure S4 Sensitivity analysis mortality. [file JGH3-4-22-s005.pdf]
