# Supplementary material for: Prophylactic endotracheal intubation in critically ill patients with upper gastrointestinal bleed: A systematic review and meta‐analysis
Source: JGH Open. 2019 May 24;4(1):22–8. doi: 10.1002/jgh3.12195 (PMC7008165; doi:10.1002/jgh3.12195)

Supplementary Figure 5: Sensitivity analysis cardiac complications and prophylactic intubation in UGIB. CI indicated confidence interval(s) and M-H, Mantel-Haenszel odds ratio.

	Prophylactic Intubation		No intubation			Odds Ratio	Odds Ratio
**Study or Subgroup**	**Events**	**Total**	**Events**	**Total**	**Weight**	**M-H, Random, 95% CI**	**M-H, Random, 95% CI**
Hayat 2017	5	100	1	100	15.9%	5.21 [0.60, 45.43]	
Perisetti 2013	6	69	2	69	0.0%	3.19 [0.62, 16.40]	
Rehman 2009	8	49	8	49	44.5%	1.00 [0.34, 2.92]	
Tang 2017	15	65	4	45	39.6%	3.08 [0.95, 9.98]	
**Total (95% CI)**		**214**		**194**	**100.0%**	**2.03 [0.80, 5.17]**	
Total events	28		13				

Heterogeneity: Tau² = 0.21; Chi² = 2.88, df = 2 (P = 0.24); I² = 31%

Test for overall effect: Z = 1.48 (P = 0.14)

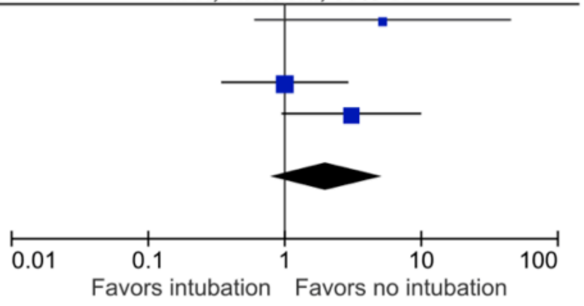

Supplement: Supplementary file 6 — Figure S5 Sensitivity analysis cardiac. [file JGH3-4-22-s006.pdf]
