# Supplementary material for: Prophylactic endotracheal intubation in critically ill patients with upper gastrointestinal bleed: A systematic review and meta‐analysis
Source: JGH Open. 2019 May 24;4(1):22–8. doi: 10.1002/jgh3.12195 (PMC7008165; doi:10.1002/jgh3.12195)

Supplementary Figure 6: Sensitivity analysis ICU LOS and prophylactic intubation in UGIB. CI indicated confidence interval(s) and M-H, Mantel-Haenszel odds ratio.

	Prophylactic Intubation			No intubation				Mean Difference
Study or Subgroup	Mean	SD	Total	Mean	SD	Total	Weight	IV, Random, 95% CI
Perisetti 2013	6	11.8	69	2	2.4	69	0.0%	4.00 [1.16, 6.84]
Rehman 2009	2.2	1.48	49	1.8	1.11	49	87.5%	0.40 [-0.12, 0.92]
Tang 2017	5.3	4	65	4.3	3.3	45	12.5%	1.00 [-0.37, 2.37]
**Total (95% CI)**			**114**			**94**	**100.0%**	**0.48 [-0.01, 0.96]**

Heterogeneity: $Tau^2 = 0.00$; $Chi^2 = 0.65$, df = 1 (P = 0.42); $I^2 = 0\%$

Test for overall effect: Z = 1.92 (P = 0.05)

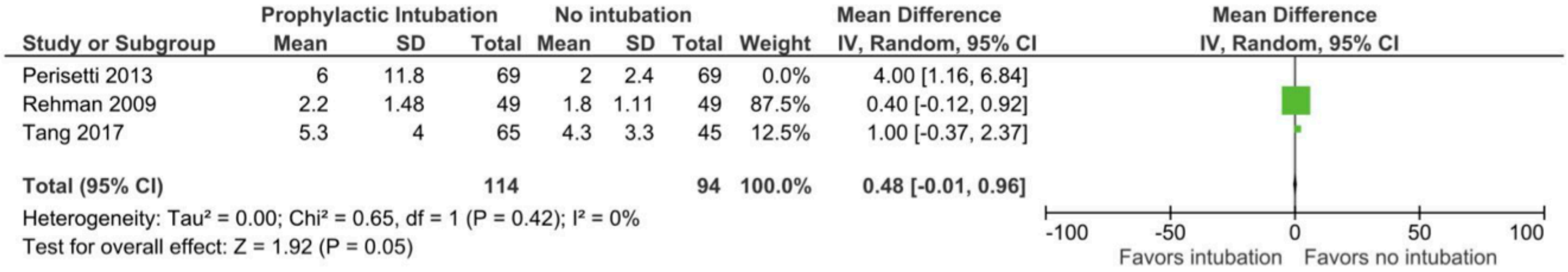

Supplement: Supplementary file 7 — Figure S6 Sensitivity analysis ICU LOS. [file JGH3-4-22-s007.pdf]
