# Supplementary material for: Prophylactic endotracheal intubation in critically ill patients with upper gastrointestinal bleed: A systematic review and meta‐analysis
Source: JGH Open. 2019 May 24;4(1):22–8. doi: 10.1002/jgh3.12195 (PMC7008165; doi:10.1002/jgh3.12195)
Supplement: Supplementary file 8 — Table S1 Baseline characteristics of studies. [file JGH3-4-22-s008.docx]

Table 1: Baseline Characteristics of Studies

|  | Study design | Inclusion criteria | Exclusion Criteria | Number of patients | | Comorbidity status | Mortality | | Cardiac complication | | Pneumonia | | Hospital Stay | | ICU stay | |
| --- | --- | --- | --- | --- | --- | --- | --- | --- | --- | --- | --- | --- | --- | --- | --- | --- |
|  |  |  |  | PI | NI |  | PI | NI | PI | NI | PI | NI | PI | NI | PI | NI |
| Tang et al (2017) | Retrospective cohort study | Variceal bleed | 1. Non-variceal bleed; 2. Intubation for pre-existing or other causes other than EGD; Hepatic encephalopathy grade 3 or higher; Pneumonia or abnormal CXR | 65 | 45 | No significant difference in the comorbidities between the two groups | 15 | 4 | 15 | 4 | 7 | 1 | 10.6 (7.9) | 8.8 (7.5) | 5.3 (4.0) | 4.3 (3.3) |
| Abdulsamad et al (2016) (abstract) | Retrospective cohort study | Any upper GI bleed | Prior intubation for any reason | 264 | 1210 | Patients with cirrhosis, ascites and altered LOC were more likely to be intubated. No difference in heart failure, chronic lung disease and kidney failure | 105 | 85 |  |  | 97 | 94 | 18 | 8 |  |  |
| Hayat et al (2017) | Retrospective cohort study; | 1. Patients > 18 years of age; 2. Brisk UGIB (hematemesis or melena with hemodynamic compromise); 3. Intubation performed solely for the purposes of airway protection and prevention of aspiration in a patient with UGIB | 1. Diagnosis of pneumonia, ARDS, MI, pulmonary edema, arrhythmia or cardiac arrest before UGIB; 2. Intubated before GI bleed; 3. EGD could not be advanced past oropharynx; 4. EGD for reasons other than GI bleed | 100 | 100 | both groups were matched for age, in hospital event, Charlton Comorbidity Index, APACHE score, Glasgow Blatchford score, history of cardiac or lung disease, history of cirrhosis, GCS, presence of shock, resuscitation with pressors, and arrhythmia 24 hours before EGD | 10 | 10 | 5 | 1 | 14 | 2 | 9.0 (4.0, 17.0) | 7.0 (4.0, 13.0) |  |  |
| Lohse et al (2015) | Prospective cohort study | 1. Patients with peptic ulcer bleeding undergoing emergency EGD under anesthesia care; 2. Patients greater than 16 years |  | 2101 | 1479 | Significant difference in shock at admission and Charlson comorbidity index - PI group had worse shock, were younger, had lower Charlson comorbidity index, had high risk ulcers and were more likely to be admitted to ICU | 238 | 125 |  |  |  |  | 8.16 (11.34) | 7.63 (13.83) |  |  |
| Perisetti et al (2013) - abstract | Retrospective cohort study | All patients with UGIB admitted to ICU who underwent EGD | Prior intubation for any reason | 69 | 69 | PI patients more likely to be younger, alcoholic, cirrhosis, lung disease, hepatic encephalopathy and sepsis | 15 | 3 | 6 | 2 | 26 | 4 | 10 (11.8) | 7 (3.8) | 6 (11.8) | 2 (2.4) |
| Rehman et al (2009) | Retrospective propensity matched case-control study | 1. UGIB admitted to ICU 2. Known cirrhosis, hematemesis or shock | 1. Refused intubation; 2. Intubated for reasons other than airway protection during UGIB; 3. Intubated prior to ICU admission | 49 | 49 | No significant difference in the comorbidities between the two groups | 7 | 10 | 8 | 8 | 9 | 5 | 6.9 (4.9, 12.7) | 5.9 (4.0, 12.3) | 2.2 (1.7, 3.7) | 1.8 (1.1, 2.6) |
| Koch et al (2006) | Retrospective cohort study | 1. Active bleeding from varices; 2. Absence of hepatic encephalopathy greater than stage 1; 3. absence of abnormal CXR before EGD; 4. absence of respiratory distress or aspiration pneumonia prior to EGD |  | 42 | 20 | No significant differences in age, etiology of liver or liver disease status | 9 | 1 |  |  | 9 | 0 | 8.2 (6) | 6.9 (7) |  |  |
