# Supplementary material for: Prophylactic endotracheal intubation in critically ill patients with upper gastrointestinal bleed: A systematic review and meta‐analysis
Source: JGH Open. 2019 May 24;4(1):22–8. doi: 10.1002/jgh3.12195 (PMC7008165; doi:10.1002/jgh3.12195)
Supplement: Supplementary file 9 — Table 2–8 Quality assessment of Individual Studies using the National Institute of Health's quality assessment tools for case–control and cohort studies. Table S9 Overall assessment of the quality of evidence presented in the systematic review using the GRADE approach. [file JGH3-4-22-s009.docx]

|  |
| --- |

Quality Assessment

Table 2: Tang et al

| Criteria | Author’s Judgement | Reason for judgement |
| --- | --- | --- |
| 1. Was the research question or objective in this paper clearly stated and appropriate? | Yes | Clearly described in study methods |
| 2. Was the study population clearly specified and defined? | Yes | Clearly described in study inclusion criteria |
| 3. Did the authors include a sample size justification? | No | No previous calculation for power performed. |
| 4. Were controls selected or recruited from the same or similar population that gave rise to the cases (including the same timeframe)? | Yes | All patients recruited were inviduals who underwent urgent bedside EGD within 12 hours of admissions for variceal hemorrhage in the ICU in a 5 year time period |
| 5. Were the definitions, inclusion and exclusion criteria, algorithms or processes used to identify or select cases and controls valid, reliable, and implemented consistently across all study participants? | Yes | Inclusion and exclusion criteria clearly defined. Data extracted by a computer database and then reviewed by study authors to ensure inclusion and exclusion criteria met. |
| 6. Were the cases clearly defined and differentiated from controls? | Yes | Patients were either prophylactically intubated or they were not intubated at all. Any patients intubated for any other reason were excluded from the study. |
| 7. If less than 100 percent of eligible cases and/or controls were selected for the study, were the cases and/or controls randomly selected from those eligible? | N/A | All eligible cases and controls participated in the study |
| 8. Was there use of concurrent controls? | Yes | This was a retrospective study so controls were from the same time period as cases. |
| 9. Were the investigators able to confirm that the exposure/risk occurred prior to the development of the condition or event that defined a participant as a case? | Yes | Any patients who had the outcomes (respiratory distress, unstable cardiopulmonary status, airway protection due to large volume blood in proxima GI tract, pneumonia or hepatic encelopathy) being examined at the study prior to EGD being performed were excluded from the study. |
| 10. Were the measures of exposure/risk clearly defined, valid, reliable, and implemented consistently (including the same time period) across all study participants? | Yes | Measure of exposure was prophylactic intubation – clearly defined. |
| 11. Were key potential confounding variables measured and adjusted statistically in the analyses? If matching was used, did the investigators account for matching during study analysis? | No | Confounding variables were measured, however, there was no adjustment statistically during the analysis. However, both groups were similar in baseline variables and there was no statistically significant difference between each. |

Table 3: Abdulsamad et al

| Criteria | Author’s Judgement | Reason for judgement |
| --- | --- | --- |
| 1. Was the research question or objective in this paper clearly stated and appropriate? | Yes | Clearly described in abstract introductions section |
| 2. Was the study population clearly specified and defined? | Yes | Clearly described in method section |
| 3. Did the authors include a sample size justification? | No | No previous calculation for power performed. |
| 4. Were controls selected or recruited from the same or similar population that gave rise to the cases (including the same timeframe)? | Yes | All patients recruited were individuals who underwent EGD in a 6 year period for upper GI bleeding. |
| 5. Were the definitions, inclusion and exclusion criteria, algorithms or processes used to identify or select cases and controls valid, reliable, and implemented consistently across all study participants? | N/A | Not clearly stated in the abstract |
| 6. Were the cases clearly defined and differentiated from controls? | N/A | Not clearly stated in the abstract |
| 7. If less than 100 percent of eligible cases and/or controls were selected for the study, were the cases and/or controls randomly selected from those eligible? | N/A | Not clearly stated in abstract |
| 8. Was there use of concurrent controls? | Yes | This was a retrospective study so controls were from the same time period as cases. |
| 9. Were the investigators able to confirm that the exposure/risk occurred prior to the development of the condition or event that defined a participant as a case? | N/A | Not clearly stated in abstract |
| 10. Were the measures of exposure/risk clearly defined, valid, reliable, and implemented consistently (including the same time period) across all study participants? | Yes | Measure of exposure was prophylactic intubation – clearly defined. |
| 11. Were key potential confounding variables measured and adjusted statistically in the analyses? If matching was used, did the investigators account for matching during study analysis? | No | Confounding variables were measured, however, there was no adjustment statistically during the analysis. However, both groups were similar in baseline variables and there was no statistically significant difference between each. |

Table 4: Hayat el al

| Criteria | Author’s Judgement | Reason for judgement |
| --- | --- | --- |
| 1. Was the research question or objective in this paper clearly stated and appropriate? | Yes | Clearly described in study methods |
| 2. Was the study population clearly specified and defined? | Yes | Clearly described in study inclusion criteria |
| 3. Did the authors include a sample size justification? | Yes | A calculation for power was performed based on the frequency of cardiopulmonary unplanned events in the first 100 subjects included in the study. The study met the numbers required for 90% power. |
| 4. Were controls selected or recruited from the same or similar population that gave rise to the cases (including the same timeframe)? | Yes | All patients recruited were individuals who underwent urgent bedside EGD in the ICU in a 3 year time period |
| 5. Were the definitions, inclusion and exclusion criteria, algorithms or processes used to identify or select cases and controls valid, reliable, and implemented consistently across all study participants? | Yes | Inclusion and exclusion criteria clearly defined. Data extracted by three human authors. |
| 6. Were the cases clearly defined and differentiated from controls? | Yes | Patients were either prophylactically intubated or they were not intubated at all. Any patients intubated for any other reason were excluded from the study. |
| 7. If less than 100 percent of eligible cases and/or controls were selected for the study, were the cases and/or controls randomly selected from those eligible? | N/A | All eligible patients included |
| 8. Was there use of concurrent controls? | Yes | This was a retrospective study so controls were from the same time period as cases. |
| 9. Were the investigators able to confirm that the exposure/risk occurred prior to the development of the condition or event that defined a participant as a case? | Yes | Any patients who had the outcomes being examined at the study prior to EGD being performed were excluded from the study. |
| 10. Were the measures of exposure/risk clearly defined, valid, reliable, and implemented consistently (including the same time period) across all study participants? | Yes | Measure of exposure was prophylactic intubation – clearly defined. |
| 11. Were key potential confounding variables measured and adjusted statistically in the analyses? If matching was used, did the investigators account for matching during study analysis? | Yes | Confounding variables were measured, and propensity score matching was done to match the prophylactic intubation group to the no prophylactic intubation group. While both groups were initially different in a number of variables, after propensity matching there were no significant differences between groups. |

Table 5: Lohse et al

| Criteria | Author’s Judgement | Reason for judgement |
| --- | --- | --- |
| 1. Was the research question or objective in this paper clearly stated and appropriate? | Yes | Clearly described in study methods |
| 2. Was the study population clearly specified and defined? | Yes | Clearly described in study inclusion criteria |
| 3. Did the authors include a sample size justification? | No | A calculation for study power was performed showing that the study was powered to detect ORs of >1.27 and <0.77. |
| 4. Were controls selected or recruited from the same or similar population that gave rise to the cases (including the same timeframe)? | Yes | All patients recruited were individuals who had peptic ulcer bleeding with emergency OGD under anesthesia between 2006 to 2013 in any hospital in Denmark. |
| 5. Were the definitions, inclusion and exclusion criteria, algorithms or processes used to identify or select cases and controls valid, reliable, and implemented consistently across all study participants? | Yes | Inclusion and exclusion criteria clearly defined. Data extracted by a computer database and then reviewed by study authors to ensure inclusion and exclusion criteria met. |
| 6. Were the cases clearly defined and differentiated from controls? | Yes | Patients were either prophylactically intubated or they had anesthetic care without airway instrumentation. Any patients intubated for any other reason were excluded from the study. |
| 7. If less than 100 percent of eligible cases and/or controls were selected for the study, were the cases and/or controls randomly selected from those eligible? | N/A | All eligible patients included |
| 8. Was there use of concurrent controls? | Yes | This was a retrospective study so controls were from the same time period as cases. |
| 9. Were the investigators able to confirm that the exposure/risk occurred prior to the development of the condition or event that defined a participant as a case? | Yes | Any patients who had the outcomes being examined at the study prior to EGD being performed were excluded from the study. |
| 10. Were the measures of exposure/risk clearly defined, valid, reliable, and implemented consistently (including the same time period) across all study participants? | Yes | Measure of exposure was prophylactic intubation – clearly defined. |
| 11. Were key potential confounding variables measured and adjusted statistically in the analyses? If matching was used, did the investigators account for matching during study analysis? | No | Confounding variables were measured, and the final odds ratios were adjusted according to the confounding variables. No matching was performed. |

Table 6: Perisetti et al

| Criteria | Author’s Judgement | Reason for judgement |
| --- | --- | --- |
| 1. Was the research question or objective in this paper clearly stated and appropriate? | Yes | Clearly described in abstract under purposes section |
| 2. Was the study population clearly specified and defined? | No | Not clearly described in abstract |
| 3. Did the authors include a sample size justification? | No | No previous calculation for power performed. |
| 4. Were controls selected or recruited from the same or similar population that gave rise to the cases (including the same timeframe)? | Yes | All patients recruited were individuals who underwent urgent bedside EGD due to a UGIB in a 13 year time period in one hospital. |
| 5. Were the definitions, inclusion and exclusion criteria, algorithms or processes used to identify or select cases and controls valid, reliable, and implemented consistently across all study participants? | No | Not clearly defined in abstract |
| 6. Were the cases clearly defined and differentiated from controls? | Yes | Patients were either prophylactically intubated or they were not intubated at all. Any patients intubated for any other reason were excluded from the study. Patients intubated during procedure or post-procedure were added to a different group. |
| 7. If less than 100 percent of eligible cases and/or controls were selected for the study, were the cases and/or controls randomly selected from those eligible? | N/A | Not defined in abstract |
| 8. Was there use of concurrent controls? | Yes | This was a retrospective study so controls were from the same time period as cases. |
| 9. Were the investigators able to confirm that the exposure/risk occurred prior to the development of the condition or event that defined a participant as a case? | N/A | Not clearly defined in abstract |
| 10. Were the measures of exposure/risk clearly defined, valid, reliable, and implemented consistently (including the same time period) across all study participants? | Yes | Measure of exposure was prophylactic intubation – clearly defined. |
| 11. Were key potential confounding variables measured and adjusted statistically in the analyses? If matching was used, did the investigators account for matching during study analysis? | No | Confounding variables were measured, however, there was no adjustment statistically during the analysis. No matching was performed between the prophylactic intubation and the non-intubation group. |

Table 7: Rehman et al

| Criteria | Author’s Judgement | Reason for judgement |
| --- | --- | --- |
| 1. Was the research question or objective in this paper clearly stated and appropriate? | Yes | Clearly described in study methods |
| 2. Was the study population clearly specified and defined? | Yes | Clearly described in study inclusion criteria |
| 3. Did the authors include a sample size justification? | Yes | A calculation was performed to detect a 25% difference in cardiopulmonary complicated between the two groups. However, the target was 50 patients per group and the study only had 49. |
| 4. Were controls selected or recruited from the same or similar population that gave rise to the cases (including the same timeframe)? | Yes | All patients recruited were from the same pool of patients |
| 5. Were the definitions, inclusion and exclusion criteria, algorithms or processes used to identify or select cases and controls valid, reliable, and implemented consistently across all study participants? | Yes | Inclusion and exclusion criteria clearly defined. Data extracted by authors of the study. |
| 6. Were the cases clearly defined and differentiated from controls? | Yes | Patients were either prophylactically intubated or they were not intubated at all. Any patients intubated for any other reason were excluded from the study. |
| 7. If less than 100 percent of eligible cases and/or controls were selected for the study, were the cases and/or controls randomly selected from those eligible? | No | Four patients in the prophylactic intubation group were not included as a matched control could not be found for them, |
| 8. Was there use of concurrent controls? | Yes | This was a retrospective study so controls were from the same time period as cases. |
| 9. Were the investigators able to confirm that the exposure/risk occurred prior to the development of the condition or event that defined a participant as a case? | Yes | Any patients who had the outcomes being examined at the study prior to EGD being performed were excluded from the study. |
| 10. Were the measures of exposure/risk clearly defined, valid, reliable, and implemented consistently (including the same time period) across all study participants? | Yes | Measure of exposure was prophylactic intubation – clearly defined. |
| 11. Were key potential confounding variables measured and adjusted statistically in the analyses? If matching was used, did the investigators account for matching during study analysis? | No | Confounding variables were measured, however, there was no adjustment statistically during the analysis. However, matching was performed and there were no significant differences between groups at baseline characteristics post matching. |

Table 8: Koch et al

| Criteria | Author’s Judgement | Reason for judgement |
| --- | --- | --- |
| 1. Was the research question or objective in this paper clearly stated and appropriate? | Yes | Clearly described in study methods |
| 2. Was the study population clearly specified and defined? | Yes | Clearly described in study inclusion criteria |
| 3. Did the authors include a sample size justification? | No | No previous calculation for power performed. |
| 4. Were controls selected or recruited from the same or similar population that gave rise to the cases (including the same timeframe)? | Yes | All patients recruited were individuals who underwent urgent bedside EGD within 12 hours of admissions for variceal hemorrhage in the ICU in a 7 year time period |
| 5. Were the definitions, inclusion and exclusion criteria, algorithms or processes used to identify or select cases and controls valid, reliable, and implemented consistently across all study participants? | Yes | Inclusion and exclusion criteria clearly defined. Data extracted by study authors to ensure inclusion and exclusion criteria met. |
| 6. Were the cases clearly defined and differentiated from controls? | Yes | Patients were either prophylactically intubated or they were not intubated at all. Any patients intubated for any other reason were excluded from the study. |
| 7. If less than 100 percent of eligible cases and/or controls were selected for the study, were the cases and/or controls randomly selected from those eligible? | N/A | All eligible patients included in the study. |
| 8. Was there use of concurrent controls? | Yes | This was a retrospective study so controls were from the same time period as cases. |
| 9. Were the investigators able to confirm that the exposure/risk occurred prior to the development of the condition or event that defined a participant as a case? | Yes | Any patients who had the outcomes being examined at the study prior to EGD being performed were excluded from the study. |
| 10. Were the measures of exposure/risk clearly defined, valid, reliable, and implemented consistently (including the same time period) across all study participants? | Yes | Measure of exposure was prophylactic intubation – clearly defined. |
| 11. Were key potential confounding variables measured and adjusted statistically in the analyses? If matching was used, did the investigators account for matching during study analysis? | No | Confounding variables were measured, however, there was no adjustment statistically during the analysis. However, both groups were similar in baseline variables except for Child Pugh score (average 1 point difference) and endoscopic therapy. |

Table 9: Assessment of overall quality of evidence using the GRADE approach (for mortality)

| Initial score based on type of evidence, study quality and risk of bias | +2: Observational studies (non-RCT)  -1: A few of the studies did not account for or accommodate confounding factors in the baseline characteristics of the two groups compared in the study. |
| --- | --- |
| Inconsistency | -1: Most studies show similar results, however, results are statistically heterogeneous |
| Indirectness | 0: No significant concern with indirectness. Two studies looked at patients with variceal bleeds only, but most look at every type of upper GI bleed. |
| Imprecision | 0: No significant concerns. Over 300 events and total number of participants over 5000. Effect estimate is quite precise. |
| Publication Bias | 0: No significant concern for publication bias |
| Upgrading factors: | +1: Large effect size |
| Final GRADE Score | +1 = Very low quality evidence |

Assessment of overall quality of evidence using the GRADE approach (for pneumonia)

| Initial score based on type of evidence, study quality and risk of bias | +2: Observational studies (non-RCT)  -1: A few of the studies did not account for or accommodate confounding factors in the baseline characteristics of the two groups compared in the study. |
| --- | --- |
| Inconsistency | 0: Most studies show similar results with no heterogeneity. |
| Indirectness | 0: No significant concern with indirectness. Two studies looked at patients with variceal bleeds only, but most look at every type of upper GI bleed. |
| Imprecision | 0: No significant concerns. Over 300 events and total number of participants over 5000. Effect estimate is quite precise. |
| Publication Bias | 0: No significant concern for publication bias |
| Upgrading factors: | +2: Very large effect size |
| Final GRADE Score | +3 = Moderate quality evidence |

Assessment of overall quality of evidence using the GRADE approach (for hospital length of stay)

| Initial score based on type of evidence, study quality and risk of bias | +2: Observational studies (non-RCT)  -1: A few of the studies did not account for or accommodate confounding factors in the baseline characteristics of the two groups compared in the study. |
| --- | --- |
| Inconsistency | 0: Most studies show similar results, with no heterogeneity. |
| Indirectness | 0: No significant concern with indirectness. Two studies looked at patients with variceal bleeds only, but most look at every type of upper GI bleed. |
| Imprecision | 0: No significant concerns. Over 300 events and total number of participants over 5000. Effect estimate is quite precise. |
| Publication Bias | 0: No significant concern for publication bias |
| Upgrading factors: | 0: None |
| Final GRADE Score | +1 = Very low quality evidence |
